# Geographic and facility variation in initial use of non-tunneled catheters for incident maintenance hemodialysis patients

**DOI:** 10.1186/s12882-016-0236-4

**Published:** 2016-02-27

**Authors:** Edward G. Clark, Ayub Akbari, Brett Hiebert, Swapnil Hiremath, Paul Komenda, Charmaine E. Lok, Louise M. Moist, Michael E. Schachter, Navdeep Tangri, Manish M. Sood

**Affiliations:** Division of Nephrology, Department of Medicine, The Ottawa Hospital and Kidney Research Centre, Ottawa Hospital Research Institute, University of Ottawa, Ottawa, ON Canada; Cardiac Sciences Program, St Boniface Hospital, Winnipeg, MB Canada; Section of Nephrology, Department of Medicine, University of Manitoba, Winnipeg, MB Canada; Division of Nephrology, Department of Medicine, Toronto General Hospital and University of Toronto, Toronto, ON Canada; Division of Nephrology, Department of Medicine, Schulich School of Medicine and Dentistry, Western University and Kidney Clinical Research Unit, London Health Sciences Centre, London, ON Canada; Royal Jubilee Hospital, Victoria, BC Canada; Seven Oaks Hospital, Winnipeg, MB Canada; The Ottawa Hospital – Riverside Campus, 1967 Riverside Drive, Ottawa, ON K1H 7 W9 Canada

**Keywords:** Vascular access, Hemodialysis, Temporary hemodialysis catheters, Epidemiology, Practice variation

## Abstract

**Background:**

Non-tunneled (temporary) hemodialysis catheters (NTHCs) are the least-optimal initial vascular access for incident maintenance hemodialysis patients yet little is known about factors associated with NTHC use in this context. We sought to determine factors associated with NTHC use and examine regional and facility-level variation in NTHC use for incident maintenance hemodialysis patients.

**Methods:**

We analyzed registry data collected between January 2001 and December 2010 from 61 dialysis facilities within 12 geographic regions in Canada. Multi-level models and intra-class correlation coefficients were used to evaluate variation in NTHC use as initial hemodialysis access across facilities and geographic regions. Facility and patient characteristics associated with the lowest and highest quartiles of NTHC use were compared.

**Results:**

During the study period, 21,052 patients initiated maintenance hemodialysis using a central venous catheter (CVC). This included 10,183 patients (48.3 %) in whom the initial CVC was a NTHC, as opposed to a tunneled CVC. Crude variation in NTHC use across facilities ranged from 3.7 to 99.4 % and across geographic regions from 32.4 to 85.1 %. In an adjusted multi-level logistic regression model, the proportion of total variation in NTHC use explained by facility-level and regional variation was 40.0 % and 34.1 %, respectively. Similar results were observed for the subgroup of patients who received greater than 12 months of pre-dialysis nephrology care. Patient-level factors associated with increased NTHC use were male gender, history of angina, pulmonary edema, COPD, hypertension, increasing distance from dialysis facility, higher serum phosphate, lower serum albumin and later calendar year.

**Conclusions:**

There is wide variation in NTHC use as initial vascular access for incident maintenance hemodialysis patients across facilities and geographic regions in Canada. Identifying modifiable factors that explain this variation could facilitate a reduction of NTHC use in favor of more optimal initial vascular access.

## Background

Non-tunneled (temporary) hemodialysis catheters (NTHCs) are the preferred initial vascular access for patients with acute kidney injury (AKI) [1]. However, for patients initiating chronic hemodialysis, NTHCs are the least optimal initial vascular access [2–4]. In this situation, the insertion of a NTHC is an additional procedure, with risks of serious complications [3], for patients who will subsequently require another procedure to establish permanent vascular access anyway. Similar to what has been well described for tunneled catheters [3], likely related to consequent central venous stenosis [1], there is some evidence to suggest that initial use of a NTHC is associated with later vascular access complications such as arteriovenous fistula (AVF) thrombosis [5].

Despite efforts aimed at improving access to and provision of pre-dialysis nephrology care to minimize the use of central venous catheters (CVCs) (tunneled CVCs and NTHCs) [6], CVCs remain the initial vascular access for up to 80 % of chronic hemodialysis patients in Canada [7]. Little is known about the use of NTHCs as previous studies have not distinguished between tunneled CVCs and NTHCs [8–10].

Regional variation in dialysis practices, either at the facility or geographic level, has been well documented in Canada and other jurisdictions [11–20]. Interventions targeting a reduction in measured practice variation have translated into improvements in patient care [18, 19]. As such, quantitating practice variation for key dialysis performance metrics, such as vascular access, could facilitate the development of programs and policies to improve care. To our knowledge, no studies have sought to examine practice variation in NTHC use in patients starting maintenance hemodialysis. In this study, we set out to measure facility and geographic variation for the initial use of NTHCs and determine the factors among patients and facilities that are associated with greater NTHC use. Identification of these factors and subsequently classifying them as either modifiable or non-modifiable would potentially help in planning and implementation of process measures designed to reduce variation.

## Methods

### Population and data sources

All adult (>18 years old) patients who started maintenance hemodialysis with a CVC from January 2001 to December 2010, captured in the Canadian Organ Replacement Registry (CORR), were included in our study. CORR is a validated registry that includes information (patient demographics, comorbidities, modality of RRT, transplantation, vascular access type and survival statistics) on all end stage kidney disease (ESKD) patients in Canada (excluding the province of Québec) who start on dialysis [21, 22]. Ethics approval was obtained from the Ottawa Health Science Network Research Ethics Board. Use of CORR was approved by the Canadian Institute for Health Information (CIHI).

### Definitions

Vascular access was defined as the vascular access used for the first hemodialysis treatment. Patients using an arteriovenous fistula (AVF) or graft (AVG) were excluded, as were patients with a co-existing AVF or AVG and CVC. CVCs are prospectively coded within CORR as either being NTHCs or tunneled CVCs. Data regarding the side (right/left) and anatomical site of catheter placement (i.e. internal jugular, subclavian or femoral) is unavailable in the registry. First visit date with a nephrologist was used to estimate the length of pre-dialysis nephrology care and categorized as either greater or less than 1 year prior to first dialysis. Distance to facility was defined as less than 50, 50 to 150 and greater than 150 kilometers, using previously published methods [23]. Individual patients and dialysis facilities were de-identified for analytic purposes. The presence of co-morbidities (angina, chronic obstructive pulmonary disease, diabetes, malignancy, serious illness, hypertension, lung disease, coronary artery bypass grafting, pulmonary edema, peripheral vascular disease, stroke, cigarette smoker, acute coronary syndrome) and laboratory values immediately prior to dialysis initiation (hemoglobin, albumin, phosphate) are captured within CORR.

Since dialysis care may vary across facilities, facility level variables were created based on clinical relevance, known association with outcomes, and quality of care indicators [24–27]. Facility-level variables included whether or not the facility offered kidney transplantation or peritoneal dialysis, mean hemoglobin and phosphate of a facility’s patients at dialysis initiation, average distance in kilometers between a facility’s patients’ primary residences and nearest dialysis facility, center size and average estimated glomerular filtration rate (eGFR) at dialysis initiation. Information on patients geographic regions was included and categorized into 12 regions as Atlantic, Northern Ontario, Greater Toronto, Eastern Ontario, Western Ontario, Manitoba, Saskatchewan, Northern Alberta, Southern Alberta, Eastern British Columbia, Vancouver and Other British Columbia regions. Facilities with 10 or less patients during the entire study period were excluded. Multiple imputation was employed for missing values [28].

### Statistical analysis

Patient and facility characteristics were compared between patients who were started on dialysis with a NTHC or tunneled CVC. Continuous variables of interest were summarized using the mean with standard deviation. Differences in characteristics were determined by the Student’s *t*-test for continuous variables and the chi-square for dichotomous variables.

Facility and geographic variation were examined using a three level, multilevel model and to assess predictors associated with a NTHC. Models were adjusted for factors thought to potentially influence decisions to initiate dialysis including facility level factors (transplantation facility, peritoneal dialysis facility, average hemoglobin and phosphate, average distance a patient resided from the nearest dialysis facility and number of HD patients treated at the facility, average eGFR at dialysis initiation), patient case mix (age, sex, body mass index, race, co-morbidities, distance to facility, serum phosphate, albumin and hemoglobin, eGFR at dialysis initiation) and calendar year. Facility and geographic variation were determined by the intra-class correlation coefficient (ICC) [29]. The intra-class correlation coefficients were calculated by dividing the variance estimate at each level by the total model variance. In our study the intra-class correlation coefficient determines the proportion of explained variation in the use of a NTHC at dialysis initiation that is due to being a member of a particular group such as patient, facility and geographic region and is reported as a percentage. Facility-level variables were centered for the facility-averages [30]. The R^2^ used to determine the percentage of variation explained at each level for the full and reduced models was determined by the Raudenbush and Byrk method, whereas the R^2^ for the total model was determined by the Snijder and Bosker method [31, 32]. We employed the SAS GLIMMIX procedure (SAS 9.2) using a logit link and employing latent variable approach at the patient level [30]. In this model the intra-class correlation coefficients were calculated for the facility and geographic regions by assuming a patient-level variance of π^2^/3 [33, 34]. To interrogate the patient-level variance assumption of π^2^/3, the ICC was further calculated using a probit link assuming a patient level variance of 1. We calculated the odds ratios for initiation with a NTHC by geographic region in a separate 2-level multilevel logistic model adjusted for patient case mix and facility-level factors listed previously. As it has been established that late nephrology referral is associated with significantly greater CVC use [35], we conducted an additional analysis limiting our cohort only to patients with pre-dialysis care > 365 days (i.e. excluding patients with shorter duration of pre-dialysis care).

Lastly, we categorized the facilities into quartiles of NTHC use and compared patient and facility characteristics associated with the highest and lowest quartiles for NTHC use. Analyses were performed using SAS version 9.2 (SAS Inc, Cary NC).

## Results

During the study period, 21,052 patients began chronic dialysis with a CVC, of which 10,183 (48.3 %) were NTHCs. Table 1 details baseline patient, facility and geographic characteristics of the study cohort according whether initial vascular access was a NTHC or a tunneled permanent HD catheter. Patients with a NTHC were less likely to be Caucasian, have shorter pre-dialysis care, have a history of vascular disease and pulmonary edema, have more laboratory abnormalities and reside further from a dialysis facility. Facilities characteristics associated with NTHC use included less likely to be a peritoneal dialysis or transplantation facility and less incident patients.

**Table 1:** Baseline patient, facility and geographic characteristics of the study cohort according to individuals who initiated dialysis with non-tunneled temporary vs. tunneled hemodialysis catheters

Characteristic			
	NTHC	tCVC	pvalue
N	10, 162 (48.3)	10,890 (51.7)	
Patient Characteristics:			
Age (± SD)	64.6 ± 15.5	64.6 ± 16.2	0.705
Sex % male	59.4	57.4	0.004
BMI (± SD)	27.3 ± 6.6	27.5 ± 6.7	0.052
Race (%)			<0.001
Caucasian	70.9	74.2	
East Asian	5.9	5.1	
Aboriginal	7.8	6.1	
South Asian	2.5	3.8	
Black	4.2	3.9	
Other	6.2	3.4	
Unknown	2.5	3.6	
Pre-dialysis care >90 days %	53.2	64.3	<0.001
Pre-dialysis care >365 days %	34.2	45.6	
Co-morbidities: %			
Angina	27.2	23.9	<0.001
Acute coronary syndrome	26.5	24.8	0.003
Pulmonary edema	34.1	29.4	<0.001
Diabetes mellitus	46.7	49.3	<0.001
Stroke	16.9	16.5	0.437
Peripheral vascular disease	23.3	21.6	0.003
Malignancy	15.1	15.3	0.788
Lung disease	14.9	13.4	0.002
Any hypertension medication(s)	81.3	85.6	<0.001
Current smoker	16.9	15.7	0.034
CABG	16.4	16.4	0.970
Serious Illness	16.0	16.2	0.708
Cause of ESRD %			<0.001
Hypertension	23.6	21.4	
Diabetes mellitus	33.8	38.2	
Glomerulonephritis	12.7	14.3	
Obstruction	2.7	2.8	
Interstitial	2.8	2.6	
Polycystic kidney disease	1.8	2.8	
Other	13.1	8.6	
Unknown	9.5	9.2	
eGFR at dialysis initiation (± SD)	9.6 ± 4.7	9.6 ± 4.4	0.435
Hemoglobin g/L (mean ± SD)	97.5 ± 17.7	98.9 ± 16.9	<0.001
Phosphatemmol/L (mean ± SD)	2.0 ± 0.8	2.0 ± 0.7	<0.001
Albumin g/L (mean ± SD)	30.3 ± 0.9	30.7 ± 0.9	<0.001
Distance from facility %			<0.001
< 50 km	73.8	76.7	
50-150	16.2	15.4	
> 150	10.0	7.9	
Facility Characteristics* (N = 61)			
Mean number of incident patients (per year)	51.3 ± 34.4	60.0 ± 42.4	<0.0001
Transplant facility %	47.7	52.3	<0.0001
Peritoneal dialysis facility %	45.9	54.1	<0.0001
Mean eGFR at dialysis initiation (± SD)	9.7 ± 1.1	9.6 ± 1.2	0.001
Mean Hemoglobin g/L (± SD)	102.0 ± 3.5	102.0 ± 3.4	0.256
Mean Phosphate mmol/L (± SD)	1.9 ± 0.1	1.9 ± 0.1	0.002
Mean distance from facility km (± SD)	56.3 ± 40.7	50.0 ± 39.6	<0.0001

*N* Number, *SD* standard deviation, *BMI* body mass index, *IQR* intra-quartile range, *CABG* coronary artery bypass graft, *ESRD* end-stage renal disease, *g/L* grams per liter, *mmol/L* millimole per liter, *AVF* arteriovenous fistula, *eGFR* estimated glomerular filtration rate in ml/min/1.73 m^2^ by the 4 variable MDRD equation, *km* kilometer

**Fig. 1:**
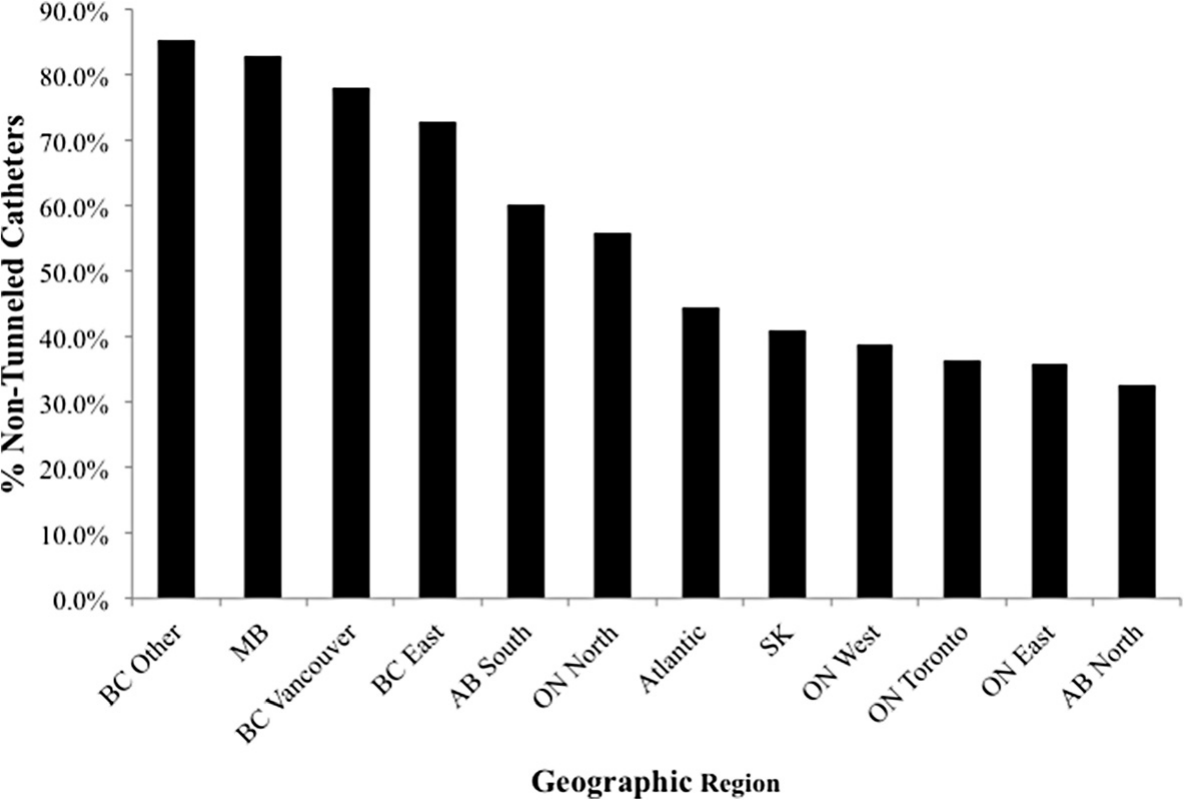
NTHC use as a percentage of crude CVC use at dialysis initiation, by geographic region. AB, Alberta; BC, British Columbia; MB, Manitoba; SK, Saskatchewan; ON, Ontario

Crude variation in NTHC use across 61 facilities ranged from 3.7 to 99.4 % and across geographic regions from 32.4 to 85.1 %. Fig. 1 shows NTHC use as a percentage of crude CVC use (NTHCs and tunneled HD catheters) across all geographic regions.

**Fig. 2:**
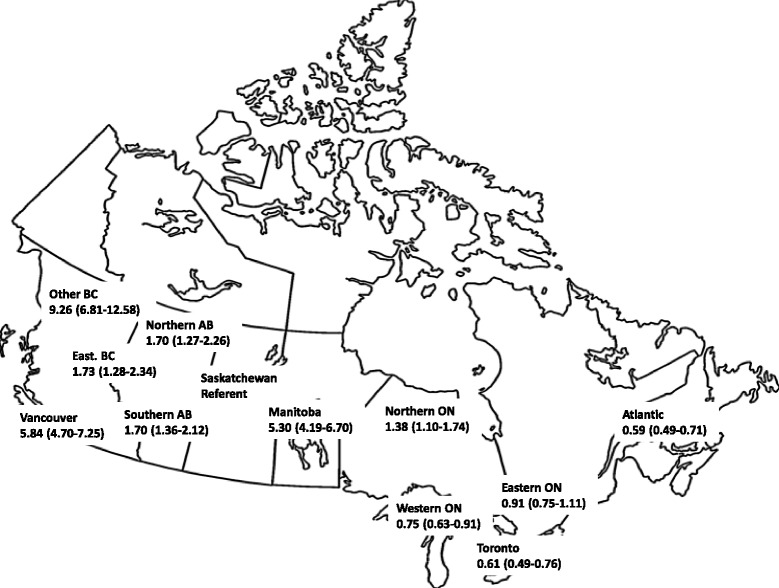
Adjusted odds ratio (with 95%CI) for initiation of hemodialysis with a NTHC across geographic regions of Canada. Adjusted for patient-level variables: age, sex, co-morbidities (CVA, angina, PVD, MI, Cancer, Pulmonary edema, COPD, DM, HTN, serious illness, CABG), BMI, laboratory values, distance from dialysis facility, cause of ESRD, race, eGFR at dialysis initiation and facility-level variables: % transplant facility, % peritoneal dialysis facility, and mean facility laboratory values, mean eGFR at dialysis initiation, mean distance from facility, mean facility size. The image was modified from http://allfreeprintable.com

Table 2 reports the unadjusted and adjusted attributable variation at the facility and geographic-level for initiation of hemodialysis with a NTHC. In the multi-level logistic regression model, the explained variation at the facility level was 43.9 % unadjusted and 40.0 % adjusted (*p* < 0.0001). Explained variation at the regional levels was 21.8 % and 34.1 % (*p* = 0.1), respectively. Similar results were observed for the subgroup of patients who received >1 year of pre-dialysis nephrology care with explained variation of 46 % and 38.1 % at the facility and regional levels, respectively (data not shown). Fig. 2 shows the adjusted odds ratios for initial NTHC use across Canada in which adjusted odds ratios ranged from 0.59 (95 % CI, 0.49-0.71) in the Atlantic region to 9.26 (95 % CI, 6.81-12.58) in BC (Other) with Saskatchewan as the reference group.

**Table 2:** Multi-level model analysis of the unadjusted and adjusted variation at the facility and geographic-level for the initiation of dialysis with a non-tunneled temporary hemodialysis catheter

	Intra-class correlation (%)	Variance estimate	Standard error	*P* value
Unadjusted				
Facility	43.9	1.44	0.30	<0.0001
Geography	21.8	0.72	0.43	0.1
Fully adjusted				
Facility	40.0	1.32	0.30	<0.0001
Geography	34.1	1.12	0.68	0.1

Geographic regions: 13; Facilities: 61; Patients:21,052

Adjusted for patient-level variables: age, sex, co-morbidities (CVA, angina, PVD, MI, Cancer, Pulmonary edema, COPD, DM, HTN, serious illness, CABG), body-mass index, laboratory values, distance from dialysis facility, cause of ESRD, race, eGFR at dialysis initiation and facility-level variables: % transplant facility, % peritoneal dialysis facility, and mean facility laboratory values, mean eGFR at dialysis initiation, mean distance from facility, mean facility size

Patient and facility characteristics for those facilities within the highest and lowest quartiles of NTHC use at dialysis initiation are compared in Table 3. In general, patients treated at facilities with the lowest quartile of NTHC use were younger, less likely to be Caucasian, had longer pre-dialysis care and lower eGFR at dialysis initiation. Conversely, patients at facilities in the highest quartile of NTHC use were more likely to have a history of pulmonary edema, stroke, cigarette smoking, PVD, serious illness, reside rurally and less likely to have diabetes, angina or ESRD due to diabetes. Facilities in the top quartile of NTHC use had fewer incident patients, and were less likely to be a transplant or peritoneal dialysis facility.

**Table 3:** Comparative patient and facility characteristics among facilities with the highest and lowest quartile of NTHC use at dialysis initiation

Characteristic			
	Lowest quartile NTHC	Highest quartile NTHC	*P* Value
N	5013(51.9)	4651(48.1)	
% with temp CVC	24.8	75.2	
Age (mean ± SD)	64.0 ± 15.5	64.8 ± 15.6	0.01
Sex % male	57.9	59.2	0.395
BMI (mean ± SD)	27.4 ± 6.7	27.6 ± 6.8	0.123
Race (%)			<0.001
Caucasian	70.9	74.2	
East Asian	5.9	5.1	
Aboriginal	7.8	6.1	
South Asian	2.5	3.8	
Black	4.2	3.9	
Other	6.2	3.4	
Unknown	2.5	3.6	
Pre-dialysis care >90 days %	61.1	56.8	<0.0001
Pre-dialysis care >365 days %	42.3	38.1	<0.0001
Co-morbidities %			
Acute coronary syndrome	26.3	25.1	0.177
Pulmonary edema	33.1	29.9	0.001
Diabetes mellitus	48.8	44.0	<0.001
Stroke	14.9	16.9	0.006
Peripheral vascular disease	21.2	23.0	0.035
Malignancy	14.9	15.9	0.150
Lung disease	13.7	14.6	0.179
Hypertension medications	85.0	77.8	<0.001
Current smoker	14.8	19.3	<0.001
CABG	13.3	14.7	0.056
Serious Illness	14.5	16.3	0.015
Angina	26.4	24.4	0.03
Cause of ESRD %			<0.001
Hypertension	22.0	23.4	
Diabetes mellitus	38.1	28.6	
Glomerulonephritis	13.6	11.3	
Obstruction	2.8	2.1	
Interstitial	2.8	1.8	
Polycystic kidney disease	2.8	2.0	
Other	8.0	20.5	
Unknown	10.0	10.3	
eGFR at dialysis initiation (mean ± SD)	9.4 ± 4.5	9.8 ± 4.6	<0.0001
Hemoglobin g/L (mean ± SD)	97.9 ± 17.3	98.2 ± 17.8	0.341
Phosphate mmol/L (mean ± SD)	2.0 ± 0.7	2.0 ± 0.7	<0.0001
Albumin g/L (mean ± SD)	31.3 ± 0.9	29.8 ± 0.9	<0.0001
Distance from facility %			<0.0001
< 50 km	75.2	72.0	<0.0001
50-150	18.6	16.6	
> 150	6.2	11.4	
Facility Characteristics			
Mean number of incident patients (per year)	78.8 ± 30.3	68.6 ± 35.4	<0.0001
Transplant facility %	31.6	28.4	0.001
Peritoneal dialysis facility %	99.6	70.5	<0.0001
eGFR at dialysis initiation (facility mean ± SD)	9.7 ± 1.1	9.6 ± 1.2	0.001
Hemoglobin g/L (facility mean ± SD)	101.6 ± 3.5	101.7 ± 4.2	0.176
Phosphate mmol/L (facility mean ± SD)	1.9 ± 0.2	1.9 ± 0.1	<0.0001
Distance from facility km (facility mean ± SD)	42.2 ± 34.7	66.9 ± 41.5	<0.0001

*NTHC* non-tunelled hemodialysis catheter, *N* Number, *eGFR* estimated glomerular filtration rate, *SD* standard deviation, *BMI* body mass index, *IQR* intra-quartile range, *CABG* coronary artery bypass graft, *ESRD* end-stage renal disease, *g/L* grams per liter, *mmol/L* millimoles per liter, *AVF* arteriovenous fistula

eGFR was estimated glomerular filtration rate in ml/min/1.73 m^2^ by the 4 variable MDRD equation, km kilometer

The extent to which patient and facility level variables were associated with incident NTHC use within the fully adjusted model are reported in Table 4. Lower number of incident patients per facility, male sex, a history of angina, pulmonary edema, lung disease, residing a further distance from a dialysis facility, higher eGFR at dialysis initiation, and higher serum phosphate were independently associated with NTHC use.

**Table 4:** Variables associated with initiation of chronic hemodialysis with a NTHC

Variables	Odds ratio	95 % Confidence interval
Level 2: Facility variables		
Transplant Facility	0.97	0.41-2.30
Peritoneal Dialysis Facility	0.89	0.33-2.44
Mean facility Hemoglobin	0.96	0.89-1.05
Mean facility Phosphate	0.14	0.01-2.30
Mean facility eGFR at dialysis initiation	0.92	0.70-1.21
Mean distance from facility	1.00	0.99-1.01
Number of Patients	1.00	1.01 - 1.15
Level 1: Patient-level variables		
Age 59 and Under vs. Age 74+	0.96	0.88-1.05
Age 60 to 73 vs. Age 74+	1.05	0.97-1.13
Male	1.10	1.03-1.18
BMI	1.00	0.99-1.00
Asian vs. Caucasian	0.91	0.78-1.06
Black vs. Caucasian	0.88	0.73-1.06
Indian Subcontinent vs. Caucasian	0.97	0.82-1.15
Aboriginal vs. Caucasian	0.93	0.80-1.07
Unknown vs. Caucasian	0.91	0.76-1.08
Other vs. Caucasian	0.78	0.64-0.94
Stroke	0.99	0.90-1.08
Angina	1.09	1.00-1.19
PVD	0.97	0.89-1.05
Acute coronary syndrome	1.06	0.97-1.16
Cancer	1.03	0.94-1.12
Pulmonary Edema	1.36	1.26-1.46
Lung disease	1.11	1.01-1.22
Diabetes	0.95	0.88-1.02
Hypertension	0.79	0.72-0.86
Serious Illness	1.07	0.98-1.17
CABG	0.98	0.89-1.08
Distance 50 to 150 vs <50	1.13	1.03-1.25
Distance >150 vs < 50	1.14	1.00-1.30
Phosphate	1.29	1.22-1.36
Albumin	0.98	0.94-1.02
Hemoglobin	0.99	0.99-0.99
eGFR at dialysis initiation	1.02	1.01-1.03
Calendar Year	0.79	0.78-0.80

## Discussion

This Canadian cohort study of over 20,000 hemodialysis patients who started dialysis between 2001 and 2010 is the first to describe patient and facility level factors associated with starting chronic hemodialysis using a NTHC. The most striking finding is the extent to which initial NTHC use varies across facilities and regions. After adjustment for patient-level factors (case-mix) and facility-level quality indicators, otherwise unspecified center-level factors accounted for 40 % of total variation and regional-level factors accounted for an additional 34 %. This is much higher than the extent to which center-level and regional-level factors explain variation for other dialysis practices in Canada such as eGFR at time of dialysis initiation (3.1 % and 0.0 %, respectively) [17] and initial use of peritoneal dialysis (9.3 % and 3.4 %, respectively) [36]. For further comparison, in studies of 173 dialysis facilities in the United States, after adjusting for case-mix, facility variation accounted for only 7.1 % of the total variation with respect to AVF use [18] and 6.7 % of the total variation with respect to dialysis adequacy [19].

Numerous factors may account for facility and regional variation in the use of NTHCs relative to tunneled CVCs. The likelihood of ‘suboptimal’ initiation of chronic dialysis, defined as having a CVC (either a tunneled catheter or NTHC) as initial vascular access maybe increased by systemic and resource limitations that affect access to timely AVF and AVG creation [8, 37, 38]. Similar considerations likely account for some of the variation observed for initial use of NTHCs versus tunneled CVCs across centers and regions. For example, centers that have better access to interventional radiology services or nephrologists and trainees capable of inserting tunneled catheters themselves may be less likely to start patients on dialysis using NTHCs. Some authors have even suggested that the need to provide training opportunities in NTHC insertion to nephrology fellows could result in the “tendency to place a non-tunneled catheter when a tunneled catheter might be more appropriate” [39]. While no jurisdiction in Canada charges patients for dialysis-related services, provider remuneration is determined at the provincial level and could influence practice patterns amongst nephrologists, vascular surgeons, intensivists and interventional radiologists accordingly.

Given that many patients begin dialysis with an NTHC in the context of severe AKI and then may become chronic HD patients in the event of non-recovery, center level variation might be accounted for to some degree by whether or not facilities have acute RRT capability. It is a limitation of this study that details regarding centers’ capability to provide acute RRT were not available to be included in the model; nonetheless, given that all regions have secondary or tertiary referral centers that provide RRT for patients with AKI in that region, regional variation is unlikely to be explained on the basis of acute RRT capability. However, regional variation in the incidence of dialysis-requiring AKI has been demonstrated in the United States [40] and similar variation in Canada could at least partially account for regional differences in the propensity for NTHC use.

Notably, similarly pronounced center-level and regional variation was observed for the subset of patients who had received > 1 year of pre-dialysis nephrology care. It has been shown that suboptimal initiation of dialysis---defined as initiation as an inpatient and/or using a CVC---frequently occurs despite an adequate duration of pre-dialysis nephrology care [19, 38, 41–43] and is associated with increased mortality [42, 44]. We suggest that, in the context of having received an adequate duration of pre-dialysis nephrology care, initiation of chronic dialysis with an NTHC is even worse than ‘suboptimal’; it is ‘least-optimal’. This is because NTHC insertion in this situation represents an additional procedure carrying risks of serious complications [3] for patients who will require a procedure to establish permanent vascular access anyway. In addition, likely related to consequent central venous stenosis [1], there is some evidence to suggest that initial use of an NTHC is associated with later vascular access complications such as AVF thrombosis [5]. Determining the causes of the unnecessary NTHC use signaled by the wide degree of practice variation could potentially lead to improved pre-dialysis care that diminishes ‘least-optimal’ dialysis starts.

A particular strength of our study is that data was derived from a large, representative cohort of incident dialysis patients from across Canada (excluding the province of Québec which does not contribute to CORR data). We utilized multi-level models and the intra-class correlation coefficient to quantitate the relative explained variation at individual levels, an analytic strategy that accounts for correlation of observations within clusters. As Canada offers universal public health care, there would be no bias due to unaffordability of health care, vascular access or dialysis services. There was little missing data on vascular access at dialysis initiation (<7 %) thus our findings are generalizable with limited selection bias.

This study has important limitations related to the use of registry data that did not include information regarding the indication for dialysis initiation including whether or not it was conducted specifically in the setting of AKI. Furthermore, while CORR registry data has been validated to have minimal risk of bias when used for clinical research [22], the coding for ‘type of catheter’ used for incident dialysis treatment has not specifically been validated. Patients with an AVF/AVG or a co-existent AVF/AVG and CVC at initial dialysis were excluded and we did not account for subsequent CVC use. A potentially high proportion of patients may experience early AVF/AVG failure and require CVC insertion. Additional limitations include the lack of data pertaining to the anatomic location of catheter insertions (e.g. internal jugular or femoral), where catheters were placed in hospitals (e.g. intensive care unit, ward, dialysis unit, radiology suite), and by which kinds of physicians (e.g. interventional radiologists, nephrologists, intensivists). A final limitation is that, while we attempted to account for facility-level complexity of care by assessing if facilities offered transplantation and/or peritoneal dialysis programs, this is not a precise way to distinguish tertiary and quaternary care facilities from others. This is particularly relevant since such centres may also be more likely to provide on-call interventional radiology service and/or acute dialysis therapy for AKI, factors that might affect the type of catheters being used (i.e. NTHC versus tunneled CVC).

## Conclusions

In conclusion, a significant proportion of the variation in initial NTHC use in chronic hemodialysis patients is explained at the facility and regional level. Given that starting dialysis with a NTHC is even less optimal than starting with a tunneled CVC, future studies are needed to determine what underpins facility-level and regional-level variation: an improved understanding of this variation could lead the way to a reduction in the frequency of ‘least-optimal’ dialysis starts using NTHCs.
